# Supplementary material for: FLYWCH transcription factors act in a LIN-42/Period autoregulatory loop during gonad migration in C. elegans
Source: bioRxiv. 2025 Jul 11:2025.07.10.664215. Preprint. [Version 1] doi: 10.1101/2025.07.10.664215 (PMC12265568; doi:10.1101/2025.07.10.664215)
Supplement: 1 [file NIHPP2025.07.10.664215v1-supplement-1.pdf]

bioRxiv preprint doi: https://doi.org/10.1101/2025.07.10.664215; this version posted July 11, 2025. The copyright holder for this preprint (which was not certified by peer review) is the author/funder, who has granted bioRxiv a license to display the preprint in perpetuity. It is made available under aCC-BY 4.0 International license.

SUPPLEMENTAL INFORMATION

bioRxiv preprint doi: https://doi.org/10.1101/2025.07.10.664215; this version posted July 11, 2025. The copyright holder for this preprint (which was not certified by peer review) is the author/funder, who has granted bioRxiv a license to display the preprint in perpetuity. It is made available under aCC-BY 4.0 International license.

Table S1. Reagent Table.

Figure S1. Loss of FLYWCH gene function causes DTC migration defects. (A) Representative image of normal gonad migration. Scale bar: 10 µm. (B) Representative image of an animal categorized as “ventral return.” (C) *flh-2(bc375)* animal categorized as “dorsal turn”. Gonad migration is initially ventralized with a later dorsal turn (n= 2/32). The gonad is outlined with a dashed white line. The DTC is marked with a yellow asterisk. (C) Percentage of samples with each phenotype for genotypes shown at bottom. Wild type, *flh-1(tm2118),* and *flh-2(tm2126)* and the double mutant *flh-1(tm2118); flh-2(tm2126)* are the same datasets as in Fig. 4B. (Wild type n=50, *nhr-85(ok2051)* n=33, *nhr-23* RNAi n=30, *flh-1(bc374)* n=32, *flh-2(bc375)* n=32, *flh-1(tm2118)* n=31, *flh-2(tm2126)* n=32, *flh-3(gk1049)* n=32, *flh-1/2* double mutant n=52, *flh-1/2* double mutant on *flh-3* RNAi n=32). The *flh-3(gk1049)* allele (a 1,200-bp deletion of the first two exons) resulted in a 18.75% defect rate while *flh-3* RNAi-treated wild type animals had a 13.33% ventral return defect rate. Statistical analysis was performed using a pairwise proportion test, with p-values adjusted for multiple comparisons via the Benjamini-Hochberg procedure, which showed that differences were not significant.

Figure S2. *lin-42* RNAi achieves efficient knockdown. (A) Skin and developing vulva of an early L4 animal fed control and *lin-42* RNAi. The white arrow points to precocious alae (n=4/10 for *lin-42* RNAi treated animals and n=0/10 for controls), cuticular features that appear at the wrong time after *lin-42* loss of function (Banerjee et al., 2005; Tennessen et al., 2006). Scale bar: 5 µm. (B) Early DTC turns observed after whole-body *lin-42* RNAi (n=3/30) and control RNAi fed animals (n=0/30) at early L3. Stage determined by vulval precursor cell number (white arrow). The gonad arm is outlined with a white dashed line. Scale bar: 5 µm (C) Early DTC turns observed after DTC-specific *lin-42* RNAi (n=2/31) and control RNAi fed animals (n=0/30) at early L3. Stage determined by vulval precursor (white arrow) cell count. The gonad arm is outlined with a white dashed line. Scale bar: 5 µm (D) Control and *lin-42* RNAi fed LIN-42::YFP stage L4.4-4.5 animals at the peak of LIN-42 expression with (right) and without (left) DIC merge showing hyp7 cells (arrows) quantified for (E). Scale bar: 5 µm. (E) Quantification of LIN-42::YFP hyp7 fluorescence intensity comparing control (n=10) and *lin-42* RNAi (n=13) in L4.4-4.5 hyp7 cells. Each data point represents an average of three hyp7 cell measurements in one animal. Mixed-effects model (REML) $F_{1, 21} = 24.33$ (****p <0.0001).

Figure S3. Transcriptional reporters of two *lin-42* promoters are dynamically expressed across cell types in L4. (A) Graphical representation of the *lin-42* locus and design of *lin-42ap::SV40-NLS::GFP::PEST*; *lin-42bp::SV40-NLS::mCherry::PEST* transcriptional reporter, each fused to a fluorescent protein coding gene with an SV40-NLS. Because of the dynamic expression we observed for LIN-42::YFP, we included PEST tags on both fluorescent proteins to promote turnover and capture signal from only newly synthesized proteins (B) Schematic illustrating the locations of the different cell types analyzed. (C) Representative images taken at L4.5 for *lin-42ap::SV40-NLS::GFP::PEST* and L4.7 for *lin-42bp::SV40-NLS::mCherry::PEST*. Both *lin-42a* and *lin-42b* are expressed in the DTC (purple), seam cells (blue), hyp7 cells (orange), and

bioRxiv preprint doi: https://doi.org/10.1101/2025.07.10.664215; this version posted July 11, 2025. The copyright holder for this preprint (which was not certified by peer review) is the author/funder, who has granted bioRxiv a license to display the preprint in perpetuity. It is made available under aCC-BY 4.0 International license.

vulval cells (red); colored dotted boxes designate focal cells in each panel. Scale bar: 5 μm. (D) Quantification of intensity of *lin-42ap::SV40-NLS::GFP::PEST* and *lin-42bp::SV40-NLS::mCherry::PEST* expression in seam cells, hyp7 cells, vulval cells, and DTC in each L4 substage. Each data point represents the average fluorescence intensity from individual animals measured by the mean intensity of three cells for each cell type except for DTC which was measured one per animal (n=8-12 per substage). Dotted lines represent SEM. In most tissue types, the temporal discrepancy between the two peaks of transcriptional activity is greater than what could reasonably be explained by differences in GFP and mCherry folding times (Balleza et al., 2018).

Figure S4. *lin-42* transcripts in populations of worms oscillate synchronously in the L3-L4 stages, while *flh-1c* rises. (A) A graph depicting average read number and SEM comparing a *lin-42a* specific exon (green), *lin-42b* specific exon (magenta), and exon shared between the two isoforms (gray) from bulk RNA-seq Illumina reads (Hendriks et al., 2014) from synchronized animals reared at 25°C, collected at one-hour intervals starting with 21 hours post-release from L1 synchronization until 36 hours post-release from L1 synchronization. (B) Data from (A) comparing *lin-42a* specific exon (green) and *lin-42b* specific exon (magenta) only. All error bars denote SEM. (C) A graph depicting average read number and SEM of *flh-1c* exons from Illumina sequencing results (Hendriks et al., 2014) from synchronized animals at one hour intervals starting with 21 hours post-release from L1 synchronization until 36 hours post-release from L1 synchronization. This bulk RNA-seq data will not reflect any DTC-specific expression dynamics, since the DTCs are a small minority of cells and these genes are not specifically expressed in the DTC.

Figure S5. *lin-42* is autorepressive in the larval skin in L4. (A) Expression intensity of *lin-42ap::SV40-NLS::GFP::PEST* and (B) *lin-42bp::SV40-NLS::mCherry::PEST* in control and lin-42 RNAi treated mutant animals in the DTC during preturn L3, turning L3, post turn L3 and each L4 substage. Each data point represents the average fluorescence intensity from individual animals measured by average of three cells for each cell type except for DTC which was measured one per animal (n=7-12 per substage). Dotted lines represent SEM. Two-way ANOVA with Tukey's multiple comparisons test for *lin-42a* ($F(27, 217) = 14.91$) and *lin-42b* ($F(27, 217) = 6.010$) (****$p < 0.0001$) (***$p < 0.001$). (C) Expression intensity of *lin-42ap::SV40-NLS::GFP::PEST* in L4.5 hyp7 cells comparing wild-type animals on control RNAi (gray, n=10), wild-type animals on *lin-42* RNAi (blue, n=16), *flh-1/2* double mutants on control RNAi (red, n=18), and flh-1/2 double mutants on *lin-42* RNAi (yellow, n=16). Two-way ANOVA with Tukey's multiple comparisons test ($F(3, 33) = 15.36$)(****$p < 0.0001$)(***$p < 0.001$)(**$p < 0.01$) (*$p < 0.05$).

Figure S6. The *lin-42b* promoter is also regulated by *flh-1/2*. (A) Expression intensity of *lin-42bp::SV40-NLS::mCherry::PEST* in wild type and *flh-1/2* double mutant animals in the DTC, seam cells, hyp7 cells, and vulval cells in each L4 substage. Each data point represents the fluorescence intensity from an individual animal measured as the mean intensity of three cells per cell type (seam, hyp7, vul), or intensity of a single DTC per animal (n=8-12 per substage). Statistics were performed using a Two-way ANOVA

bioRxiv preprint doi: https://doi.org/10.1101/2025.07.10.664215; this version posted July 11, 2025. The copyright holder for this preprint (which was not certified by peer review) is the author/funder, who has granted bioRxiv a license to display the preprint in perpetuity. It is made available under aCC-BY 4.0 International license.

with Tukey's multiple comparisons test on the DTC (F (21, 183) = 15.45), seam cells (F (21, 184) = 20.55), hyp7 cells (F (21, 184) = 20.74), and vulva (F (21, 184) = 15.76)(*p <0.05)(**p <0.01)(***p <0.001)(****p <0.0001). (B) Representative images from three staged specimens each of *lin-42bp::SV40-NLS::mCherry::PEST* in otherwise wild-type controls. The gonad is outlined with a white dotted line, and the DTC is marked with a white arrow. Scale bar: 5 µm. (C) Quantification of fluorescence intensity of *lin-42bp::SV40-NLS::mCherry::PEST* in otherwise wildtype (gray), and *flh-1/2* (purple) during each L3 DTC turning stage. Values are normalized to post-turn mean expression for each genotype. Two-way ANOVA with Tukey's multiple comparisons test (F (5, 37) = 7.442) (*p <0.1). (D) Expression intensity of *lin-42bp::SV40-NLS::mCherry::PEST* in control and *lin-42* RNAi treated animals in the DTC during preturn L3, turning L3, post turn L3 and each L4 substage. Each data point represents the average fluorescence intensity from individual animals measured by average of three cells for each cell type except for DTC which was measured one per animal (n=7-12 per substage). Dotted lines represent SEM. Statistics were performed using a Two-way ANOVA with Tukey's multiple comparisons test (F (27, 203) = 2.676), and found no significant difference of *lin-42b* expression in the DTC after *lin-42* RNAi.

Figure S7. Yeast one-hybrid hits from the *lin-42b* promoter screen. (A) A graphical representation of the *lin-42* locus and the region used as bait used for the *lin-42b* yeast one-hybrid screen. (B) List of *lin-42b* bait-binding transcription factor genes, including their dynamic transcription factor class and phase (from Hendriks et al., 2014).
